# Association Analysis of Insulin Resistance Metabolic Score (METS‐IR) and Gestational Diabetes Mellitus: Based on National Health and Nutrition Examination Survey Database From 2007 to 2018

**DOI:** 10.1002/edm2.70062

**Published:** 2025-05-30

**Authors:** Hou Wenxuan, Xu Lingyun, Tang Yujie, Zhang Ting, Han Zhen, Luo Xiao, Yang Zhao

**Affiliations:** ^1^ Department of Obstetrics and Gynecology The First Affiliated Hospital of Xi'an Jiaotong University Xi'an China; ^2^ Department of Infectious Diseases The First Affiliated Hospital of Xi'an Jiaotong University Xi'an China; ^3^ Department of Obstetrics and Gynecology Northwest Women's and Children's Hospital Xi'an China; ^4^ Department of Physiology and Pathophysiology School of Basic Medical Sciences, Xi'an Jiaotong University Health Science Center Xi'an China

**Keywords:** blood glucose, GDM, insulin resistence, METS‐IR

## Abstract

**Objective:**

This study focused on the association of the Insulin resistance metabolic score (METS‐IR) with the risk of gestational diabetes mellitus (GDM) using data from the National Health and Nutrition Examination Survey (NHANES).

**Methods:**

Data from 6 cycles of NHANES (2007–2018) were analysed. Weighted logistic regression models were constructed to explore the relationship between METS‐IR and GDM. Stratified and subgroup analyses with adjustment for confounding factors were carried out to explore the association between METS‐IR and GDM.

**Results:**

A total of 5189 samples were analysed. Based on the weighted logistic regression model, Ln(METS‐IR) was positively associated with GDM with full adjustment (OR = 1.94, 95% CI 1.08–3.46, *p* < 0.005). After transferring Ln(METS‐IR) into a categorical variable by quartiles, the positive connection between Ln(METS‐IR) and GDM was still observed in the higher Ln(METS‐IR) group compared to the lowest Ln(METS‐IR) interval (OR of 1.86, 1.76 for participants in the Q3(3.73, 3.93) and Q4(3.93, 4.83) quartile, respectively, *p* < 0.05). The threshold effect model showed that when Ln(METS‐IR) ≤ 4, the positive correlation between Ln(METS‐IR) and GDM was more significant (β = 2.69, 95% CI 1.55–4.67, *p* = 0.0004). The area under the ROC curves of Ln(METS‐IR) for GDM was 0.603, suggesting Ln(METS‐IR) a more systematic predictor for GDM. Specifically, the OR and 95% CIs of GDM for women above high school in the Q2, Q3, and Q4 quartiles were 2.05 (1.04, 4.02), 3.41 (1.72, 6.78) and 2.78 (1.55, 4.99), respectively.

**Conclusion:**

METS‐IR in women elevates the likelihood of GDM occurrence. METS‐IR serves as a comprehensive alternative to HOMA‐IR rather than HbA1c and non‐based insulin level to predict GDM.

## Graphical abstract

The results showed a strong association between METS‐IR and GDM. Considering that METS‐IR includes biochemical indicator parameters related to adipose tissue metabolism, it is suggested that METS‐IR is a more systematic predictor of GDM.

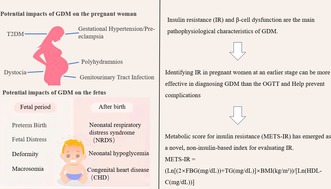

## 1 Introduction

Gestational diabetes mellitus (GDM) complicates 6%–8% of pregnancies and significantly increases the risk of short‐ and long‐term adverse outcomes for both the mothers with GDM and their offspring, including macrosomia, large‐for‐gestational‐age infants, obesity, and type 2 diabetes [1, 2]. Globally, due to improvements in people's living standards and changes in lifestyle such as sedentary behaviour, the number of obese women of childbearing age is on the rise, and the age of mothers is parallelly increasing. Thus, GDM has become one of the common complications during pregnancy [3]. The current gold standard for diagnosing GDM is the Oral Glucose Tolerance Test (OGTT) conducted between the 24th and 28th weeks of pregnancy, with glycated haemoglobin (HBA1C) also serving as an auxiliary diagnostic tool for GDM. Once diagnosed with GDM, a balanced diet and appropriate exercise to manage blood glucose levels are used in reducing the incidence of hyperglycaemia and lowering the risk of complications for both the mother and the offspring [4]. More seriously, some studies have shown that even if the blood glucose levels of pregnant women with GDM at 24–28 weeks are controlled within the normal range, the risks of epigenetic changes and long‐term metabolic abnormalities for their offspring still persist [5]. Therefore, earlier identification and intervention of GDM are crucial to safeguard the health of both GDM pregnant women and their offspring.

Insulin resistance (IR) and β‐cell dysfunction are the main pathophysiological characteristics of GDM. Insulin sensitivity undergoes significant changes throughout pregnancy, constantly adapting to the energy demands of both the mother and fetus. Therefore, identifying IR in pregnant women at an earlier stage can be more effective in diagnosing GDM than the OGTT [6]. The gold standard for assessing IR is the hyper‐insulinemic euglycemic clamp, but its high cost and invasiveness limit its clinical application during pregnancy [7]. Metabolic score for insulin resistance (METS‐IR) has emerged as a novel, non‐insulin‐based index for evaluating IR and has gained attention from researchers in recent years [8, 9]. METS‐IR was calculated through simple anthropometric measurements and biochemical parameters including fasting plasma glucose (FPG), triglycerides (TG), body mass index (BMI) and high‐density lipoprotein cholesterol (HDL‐c), into a formula, Ln [(2 × FBG + TG) × BMI]/Ln(HDL‐C), to quantify IR severity [10, 11]. Increasing studies have shown associations between METS‐IR and metabolic disorders like T2DM, metabolic syndrome, hypertension and coronary artery disease [12, 13]. However, the relationship between METS‐IR and GDM has never been studied.

Based on this point, this study utilised data from the National Health and Nutrition Examination Survey (NHANES) for 6 cycles from 2007 to 2018. The association of METS‐IR with GDM was analysed by establishing a weighted logistic regression model, considering factors such as age, race, BMI, smoking, history of hypertension, a history of delivering an infant weighing 9 pounds or more, searching for the optimal replacement indices for IR and identifying high‐risk groups for IR.

## 2 Methods

### 2.1 Study Design and Participants

Data were obtained from the NHANES database (http://www.cdc.gov/nchs/nhanes.htm), which is a representative, stratified, multi‐stage cross‐sectional survey designed and conducted by the National Center for Health Statistics (NCHS) of the United States. The freely accessible database provides health and nutrition statistics on the non‐institutionalised people in the USA. All participants provided informed consent, which was approved by the Institutional Review Board of the NCHS. Detailed statistics are shown at https://www.cdc.gov/nchs/nhanes/.

**FIGURE 1:**
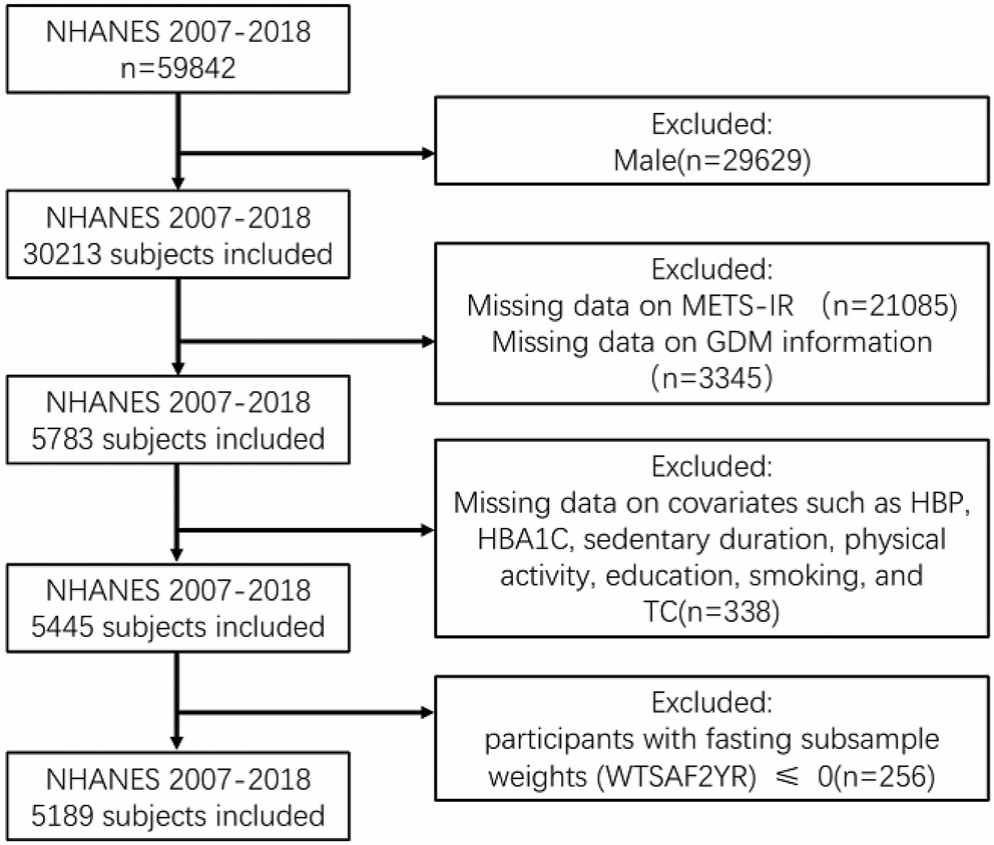
Flow chart of the participants.

For this study, 59,842 participants were involved in six NHANES cycles from 2007 to 2018. The participants included in this study included both women who had at least one live birth and those who are currently pregnant at week 28 or above. Thus, 29,629 male participants, 21,085 female participants with missing information on METS‐IR, 3345 female participants with missing information on GDM, as well as 17,659 female participants with missing covariates (HBP, HBA1C, sedentary duration, physical activity, education, smoking and TC) were excluded. In addition, we excluded 256 female participants whose weight of the fasting subsample is less than or equal to 0. Eventually, 5189 representative participants were enrolled in the study (Figure 1).

### 2.2 Metabolic Score for Insulin Resistance

METS‐IR was calculated by the arithmetic formula:

METS‐IR = (Ln[(2 × FBG(mg/dL)) + TG(mg/dL)] × BMI(kg/m^2^))/[Ln(HDL‐C(mg/dL))] [10, 11]. FBG, TG and HDL‐C were assessed at morning blood samples from participants after an 8.5‐h overnight fast. BMI was calculated as weight (kg) divided by height (m^2^).

Before statistical analysis, METS‐IR was log‐transformed to obtain Ln(METS‐IR) to better demonstrate the relationship between METS‐IR and GDM.

### 2.3 Gestational Diabetes Mellitus

The GDM diagnosis data were measured according to personal interviews: (1) item RHQ162, ‘During pregnancy, were you ever told by a doctor or other health professional that you had diabetes, hyperglycemia, or gestational diabetes?’; (2) item DIQ175S, ‘Whether GDM is your risk for diabetes or prediabetes?’. Women participants who answered ‘yes’ to either of these two questions were defined as possessing a GDM history [14, 15]. (3) Item DIQ010, ‘Other than during pregnancy, have you ever been told by a doctor or health professional that you have diabetes or sugar diabetes?’ Women who answered ‘yes’ were defined as having been diagnosed with diabetes.

### 2.4 Covariates

In the current study, we identified the demographic variables as potential confounding factors: age, race, the ratio of family income to poverty (PIR), high blood pressure (HBP), HBA1C (%), direct HDL‐C (mg/dL) and total cholesterol (mg/dL); the questionnaire data included education level, smoking status (smoking at least 100 cigarettes over the lifetime), sedentary duration (minutes/day) and a history of delivering an infant weighing 9 pounds or more.

### 2.5 Statistical Analysis

The study participants were classified into four groups based on their Ln(METS‐IR) quartile: low (Q1: 2.84, 3.53), medium1 (Q2: 3.53–3.73), medium2 (Q3: 3.73–3.93) high (Q4: 3.93–4.83) METS‐IR groups. Continuous variables were expressed as means ± standard deviations, while categorical variables were expressed as frequencies with percentages. Multiple Logistic Regression was used to analyse the differences in categorical or continuous variables among the Ln(METS‐IR) quartile groups.

Multivariable logistic regression models were used to examine the relationship between the METS‐IR and GDM with odds ratios (ORs) and 95% confidence intervals (CIs). Model 1 was unadjusted, and model 2 was adjusted for age and race. Model 3 was further adjusted for education level, PIR, HBP, total cholesterol, smoking, physical activity, sedentary duration and a history of delivering an infant weighing 9 pounds or more.

The employment of smooth curve fitting and generalised additive models allowed for the examination of whether the independent variable was segmented into distinct intervals, thereby assessing the non‐linear association between the independent variable and GDM. We will conduct further investigation of the diagnostic effectiveness of the METS‐IR using a receiver operating characteristic (ROC) curve.

Two‐piecewise regression model was employed to analyse the nonlinear relationship between Ln(METS‐IR) and GDM, and the inflection point was determined through the likelihood ratio test.

Empower(R) (X&Y Solutions Inc., MA, USA) and Stata (version 14.0) were used for statistical analysis. Statistical significance was set at *p*‐value < 0.05.

## 3 Results

### 3.1 Baseline Characteristics of Participants

The baseline characteristics of 5189 participants by the history of GDM were shown as Table 1. There were 417 cases of GDM among these participants. The baseline results showed that the mean METS‐IR value for patients with GDM was 3.83 (0.29), significantly higher than the mean METS‐IR value of 3.74 (0.29) for non‐GDM patients (*p* < 0.001). Comparison between participants with and without GDM revealed significant statistical differences (*p* < 0.05) in age, HBA1C, race, education level, a history of delivering an infant weighing 9 pounds or more and METS‐IR.

**TABLE 1:** Comparison of clinical and biochemical characteristics between non‐GDM and GDM groups.

Characters	Total	Non‐GDM	GDM	*p*
Overrall	5189	4772	417	
Age (years), mean (SD)	52.86 (16.48)	53.47 (16.66)	45.88 (12.21)	< 0.001
**Age (%)**				< 0.001
≤ 50	44.92	2056 (43.08)	275 (65.95)	
> 50	55.08	2716 (56.92)	142 (34.05)	
**Race (%)**				0.0081
Mexican American	848 (16.35)	8.06 (6.67,9.71)	12.30 (9.42,15.91)	
Other hispanic	619 (11.93)	5.77 (4.72,7.04)	6.08 (4.08,8.99)	
Non‐hispanic White	2151 (41.45)	67.39 (64.18,70.44)	60.52 (53.44,67.18)	
Non‐hispanic Black	1055 (20.33)	11.92 (10.27,13.79)	12.01 (9.15,15.61)	
Other race	516 (9.94)	6.85 (5.84,8.02)	9.09 (6.52,12.54)	
**Education level (%)**				0.020
Less than high school	1363 (26.27)	1248 (26.15)	115 (27.58)	
High school or GED	1194 (23.01)	1121 (23.49)	73 (17.51)	
Above high school	2632 (50.72)	2403 (50.36)	229 (54.92)	
**PIR (%)**				0.839
≤ 1.3	1610 (31.03)	1481 (31.04)	129 (30.94)	
> 1.3, ≤ 3.5	2329 (44.88)	2137 (44.78)	192 (46.04)	
> 3.5	1250 (24.09)	1154 (24.18)	96 (23.02)	
**HBP (%)**				0.671
No	3010 (58.01)	2764 (57.92)	246 (58.99)	
Yes	2179 (41.99)	2008 (42.08)	171 (41.01)	
**Smoking (%)**				0.988
No	3262 (62.86)	3000 (62.87)	262 (62.83)	
Yes	1927 (37.14)	1772 (37.13)	155 (37.17)	
**Physical activity (%)**				0.384
None	3020 (58.2)	2771 (58.07)	249 (59.71)	
Moderate	1469 (28.31)	1348 (28.25)	121 (29.02)	
Vigorous	700 (13.49)	653 (13.68)	47 (11.27)	
**A history of delivering an infant weighing 9 pounds or more (%)**				< 0.001
No	4248 (81.87)	3937 (82.50)	311 (74.58)	
Yes	941 (18.13)	835 (17.50)	106 (25.42)	
**Sedentary duration (%)**				0.678
≤ 450	3637 (70.09)	3341 (70.01)	296 (70.98)	
> 450	1552 (29.91)	1431 (29.99)	121 (29.02)	
HBA1C (%), mean (SD)	5.82 (1.08)	5.77 (1.00)	6.35 (1.64)	< 0.001
TC (mg/ml), mean (SD)	196.40 (42.04)	196.40 (41.48)	196.34 (48.01)	0.978
Ln(METS‐IR), mean (SD)	3.74 (0.29)	3.73 (0.28)	3.83 (0.29)	< 0.001

Thus, the study population was subsequently divided into four groups by the Ln(METS‐IR) quartiles, including Q1 (2.84, 3.53), Q2(3.53, 3.73), Q3 (3.73,3.93) and Q4 (3.93,4.83) (Table 2). As the quartiles of Ln(METS‐IR) increased, the HBA1C, HBP, sedentary duration, a history of delivering an infant weighing 9 pounds or more (%), smoking (%) and GDM (%) showed a gradual increase. Interestingly, the percent of participants under the age of 50 decreased as Ln(METS‐IR) increased, while at Q4 (3.93, 4.83), the percent of participants was higher than that of Q2(3.53, 3.73) and Q3 (3.73,3.93). As for the percent of participants over the age of 50, Q2(3.53, 3.73) of Ln(METS‐IR) account for the highest level. In addition, PIR, race, education level and sedentary duration were statistically significant, although these covariates showed a similar tendency as age with the quartiles of METS‐IR increasing.

**TABLE 2:** Baseline characteristics of the study population stratified by quartiles of Ln(METS‐IR) value.

Variable	Ln(METS‐IR)				*p*
	Q1 (2.84, 3.53)	Q2 (3.53, 3.73)	Q3 (3.73, 3.93)	Q4 (3.93, 4.83)	
Age (years), mean(SE)	50.71 (0.59)	52.96 (0.53)	52.73 (0.56)	50.10 (0.45)	0.0002
**Age (%)**	0.0008				
≤ 50	52.10 (48.49, 55.69)	44.19 (40.58, 47.86)	44.55 (41.12, 48.03)	50.97 (47.38, 54.54)	
> 50	47.90 (44.31, 51.51)	55.81 (52.14, 59.42)	55.45 (51.97, 58.88)	49.03 (45.46, 52.62)	
**Race (%)**	< 0.0001				
Mexican American	4.90 (3.88, 6.18)	8.89 (7.18, 10.96)	9.84 (7.86, 12.25)	10.60 (8.45, 13.23)	
Other hispanic	4.65 (3.55, 6.06)	6.04 (4.63, 7.83)	7.17 (5.75, 8.92)	5.55 (4.30, 7.13)	
Non‐hispanic White	73.40 (69.85, 76.66)	66.55 (62.74, 70.16)	62.99 (58.60, 67.17)	63.21 (58.77, 67.43)	
Non‐hispanic Black	7.51 (6.08, 9.23)	11.25 (9.23, 13.64)	13.42 (11.19, 16.01)	16.49 (13.86, 19.51)	
Other race (including multi‐racial)	9.55 (7.79, 11.66)	7.27 (5.70, 9.24)	6.58 (4.80, 8.98)	4.15 (3.15, 5.45)	
**Education level (%)**	< 0.0001				
Less than high school	11.66 (9.91, 13.66)	16.52 (14.17, 19.17)	19.56 (16.93, 22.50)	22.71 (20.21, 25.41)	
High school or GED	19.87 (17.49, 22.49)	24.66 (21.46, 28.16)	27.97 (24.79, 31.40)	25.35 (22.33, 28.62)	
Above high school	68.47 (64.98, 71.76)	58.82 (54.65, 62.87)	52.46 (48.86, 56.04)	51.95 (48.35, 55.52)	
**PIR**	< 0.0001				
≤ 1.3	17.69 (15.06, 20.66)	21.63 (18.46, 25.18)	22.37 (19.59, 25.42)	28.39 (25.40, 31.57)	
> 1.3 and ≤ 3.5	36.47 (32.90, 40.18)	41.49 (37.44, 45.66)	47.53 (44.10, 50.98)	45.20 (41.73, 48.72)	
> 3.5	45.85 (41.67, 50.08)	36.87 (32.84, 41.10)	30.10 (26.24, 34.26)	26.41 (22.46, 30.78)	
**HBP (%)**					< 0.0001
No	77.46 (74.26, 80.37)	65.85 (62.37, 69.16)	57.54 (53.40, 61.57)	44.19 (40.42, 48.04)	
Yes	22.54 (19.63, 25.74)	34.15 (30.84, 37.63)	42.46 (38.43, 46.60)	55.81 (51.96, 59.58)	
**Smoking**	0.1014				
No	59.89 (56.47, 63.21)	63.23 (59.07, 67.19)	59.87 (56.40, 63.24)	57.11 (54.12, 60.04)	
Yes	40.11 (36.79, 43.53)	36.77 (32.81, 40.93)	40.13 (36.76, 43.60)	42.89 (39.96, 45.88)	
**Physical activity (%)**	< 0.0001				
None	38.46 (34.55, 42.52)	52.01 (48.11, 55.87)	57.67 (54.01, 61.26)	64.40 (60.44, 68.17)	
Moderate	33.97 (29.94, 38.25)	32.79 (28.99, 36.82)	30.50 (27.33, 33.87)	26.85 (23.70, 30.25)	
Vigorous	27.57 (23.80, 31.69)	15.20 (12.61, 18.22)	11.82 (9.26, 14.98)	8.75 (6.67, 11.40)	
**A history of delivering an infant weighing 9 pounds or more (%)**	< 0.0001				
No	86.93 (84.82, 88.78)	83.94 (81.19, 86.35)	80.54 (77.57, 83.19)	75.96 (72.46, 79.14)	
Yes	13.07 (11.22, 15.18)	16.06 (13.65, 18.81)	19.46 (16.81, 22.43)	24.04 (20.86, 27.54)	
**Sedentary duration (%)**					0.0047
≤ 450 min	70.92 (67.61, 74.02)	66.59 (62.48, 70.46)	67.69 (63.67, 71.46)	61.49 (57.19, 65.62)	
> 450 min	29.08 (25.98, 32.39)	33.41 (29.54, 37.52)	32.31 (28.54, 36.33)	38.51 (34.38, 42.81)	
**HBA1C (%), mean(SE)**	5.38 (0.01)	5.54 (0.03)	5.74 (0.03)	6.09 (0.04)	< 0.0001
**TC (mg/dl), mean(SE)**	196.56 (1.64)	200.78 (1.52)	199.97 (1.65)	193.10 (1.92)	0.0026
**GDM (%)**					< 0.0001
No	95.34 (93.22, 96.82)	94.02 (91.99, 95.56)	90.77 (88.03, 92.94)	88.34 (86.01, 90.33)	
Yes	4.66 (3.18, 6.78)	5.98 (4.44, 8.01)	9.23 (7.06, 11.97)	11.66 (9.67, 13.99)	

### 3.2 The Association Between Ln(METS‐IR) and GDM

**FIGURE 2:**
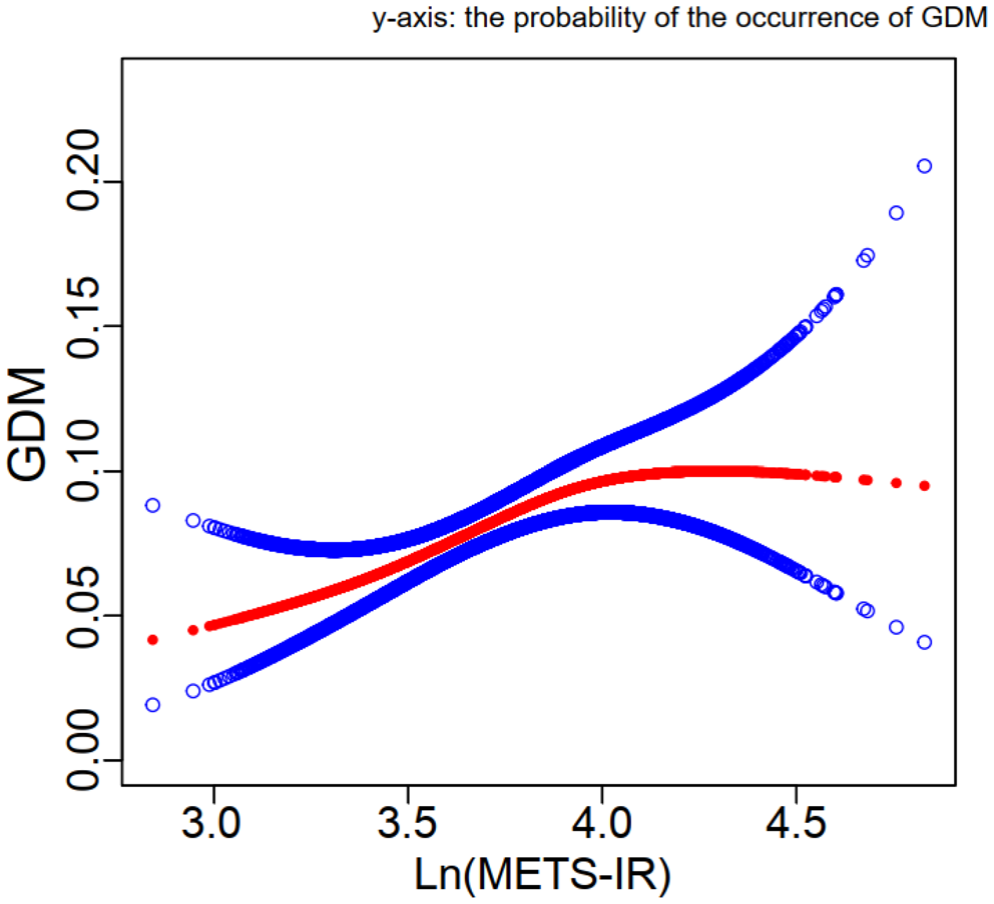
Smooth curve fitting for the relations between Ln(METS‐IR) and Probability of GDM occurrence (%). Analysis was adjusted for age, race, education level, the ratio of family PIR, high blood pressure, total cholesterol, smoking, physical activity, sedentary duration and a history of delivering an infant weighing 9 pounds or more. The solid red and blue lines symbolise the probability and corresponding 95% confidence intervals, respectively.

GDM was defined as the dependent variable, and the above‐mentioned relevant indicators (Tables 1 and 2) as independent variables. Following the adjustment for potential confounders including age, race, education level, PIR, HBP, TC, smoking, physical activity, sedentary duration and a history of delivering an infant weighing 9 pounds or more, multivariable logistic regression model 3 revealed OR (95% CI) of 1.29, 1.86 and 1.76 for participants in the Q2, Q3 and Q4 quartiles, respectively (Table 3). Smooth curve fitting for the relationship between Ln(METS‐IR) and GDM was nonlinear and U‐shaped (*p* < 0.001; Figure 2 and Table 4).

**TABLE 3:** Association between METS‐IR and GDM, weighted.

GDM	GDM OR (95% CI), *p*‐value					
	Ln(METS‐IR)					
	Per 1 increment	Q1 (2.84, 3.53)	Q2 (3.53, 3.73)	Q3 (3.73, 3.93)	Q4 (3.93, 4.83)	*p* for trend
Model 1	3.77 (2.36, 6.03) < 0.0001	Ref.	1.30 (0.78, 2.16) 0.3119	2.08 (1.24, 3.50) 0.0071	2.70 (1.74, 4.19) < 0.0001	< 0.0001
Model 2	3.80 (2.37, 6.11) < 0.0001	Ref.	1.41 (0.84, 2.36) 0.2019	2.28 (1.31, 3.97) 0.0046	2.82 (1.78, 4.46) < 0.0001	< 0.0001
Model 3	1.94 (1.08, 3.46) 0.0285	Ref.	1.29 (0.75, 2.20) 0.3563	1.86 (1.04, 3.32) 0.0407	1.76 (1.03, 3.00) 0.0417	0.0160

*Note:* Model 1: unadjusted. Model 2: adjusted for age and race. Model 3: adjusted for age, race, education level, the ratio of family PIR, high blood pressure, total cholesterol, smoking, physical activity, sedentary duration and A history of delivering an infant weighing 9 pounds or more.

Abbreviations: 95% CI, 95% confidence interval; OR, odds ratio.

**TABLE 4:** Threshold effect analysis of Ln(METS‐IR) on GDM using the two‐piecewise regression model.

GDM	HR (95% CI) *p*‐value
Model 1	
One line	1.81 (1.21, 2.71) 0.0038
Model II	
Inflection value	4
< Threshold value	2.69 (1.55, 4.67) 0.0004
≥ Threshold value	0.55 (0.17, 1.83) 0.3305
*p* for log‐likelihood ratio test	0.20 (0.05, 0.91) 0.0365

Furthermore, the threshold effect is analysed. The threshold effect model showed that when Ln(METS‐IR) ≤ 4, the positive correlation between Ln(METS‐IR) and GDM was more significant (β = 2.69, 95% CI 1.55–4.67, *p* = 0.0004). When Ln(METS‐IR) > 4, the significance decreases (β = 0.55, 95% CI 0.17–1.83, *p* = 0.3305).

### 3.3 Diagnostic Efficacy of Ln(METS‐IR) for GDM

**FIGURE 3:**
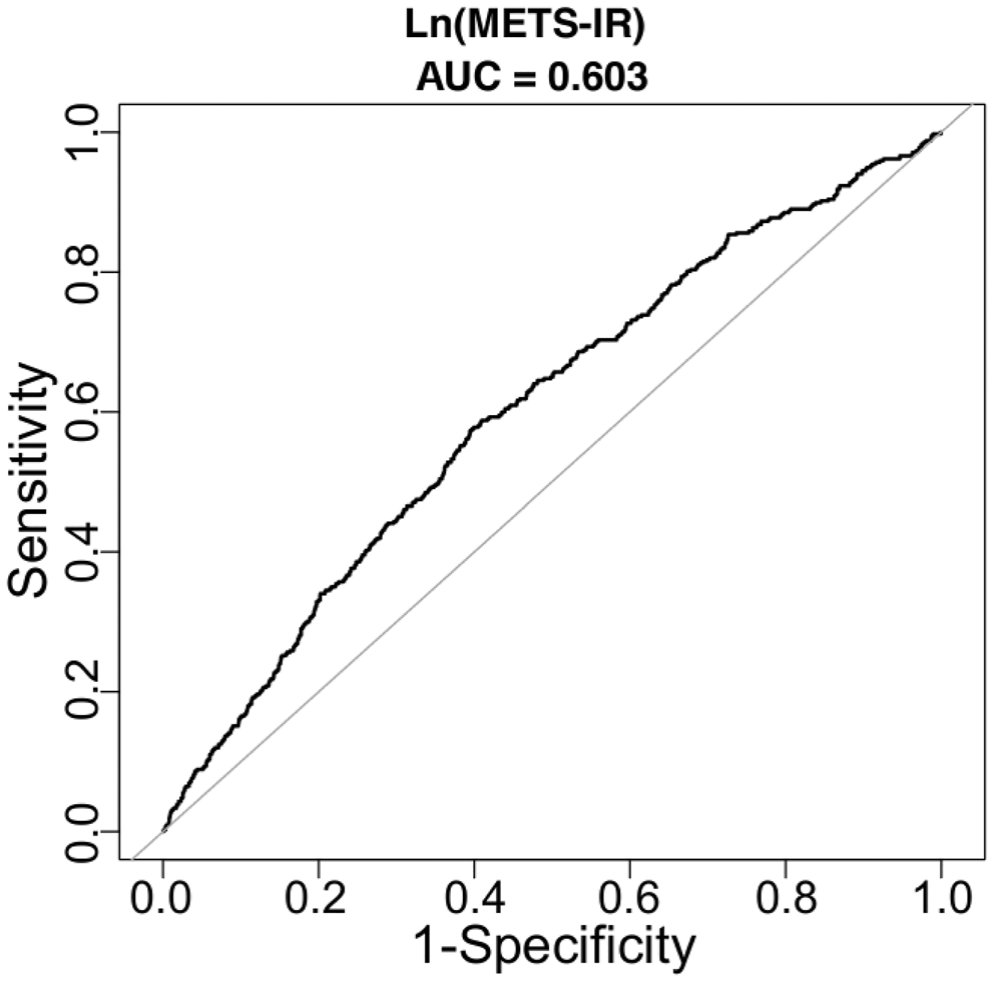
Diagnostic efficacy of METS‐IR for GDM.

Finally, we found the area under the ROC curves of Ln(METS‐IR) for GDM was 0.603 (95% CI 0.5746–0.6311) (Figure 3).

### 3.4 Subgroup Analysis

**FIGURE 4:**
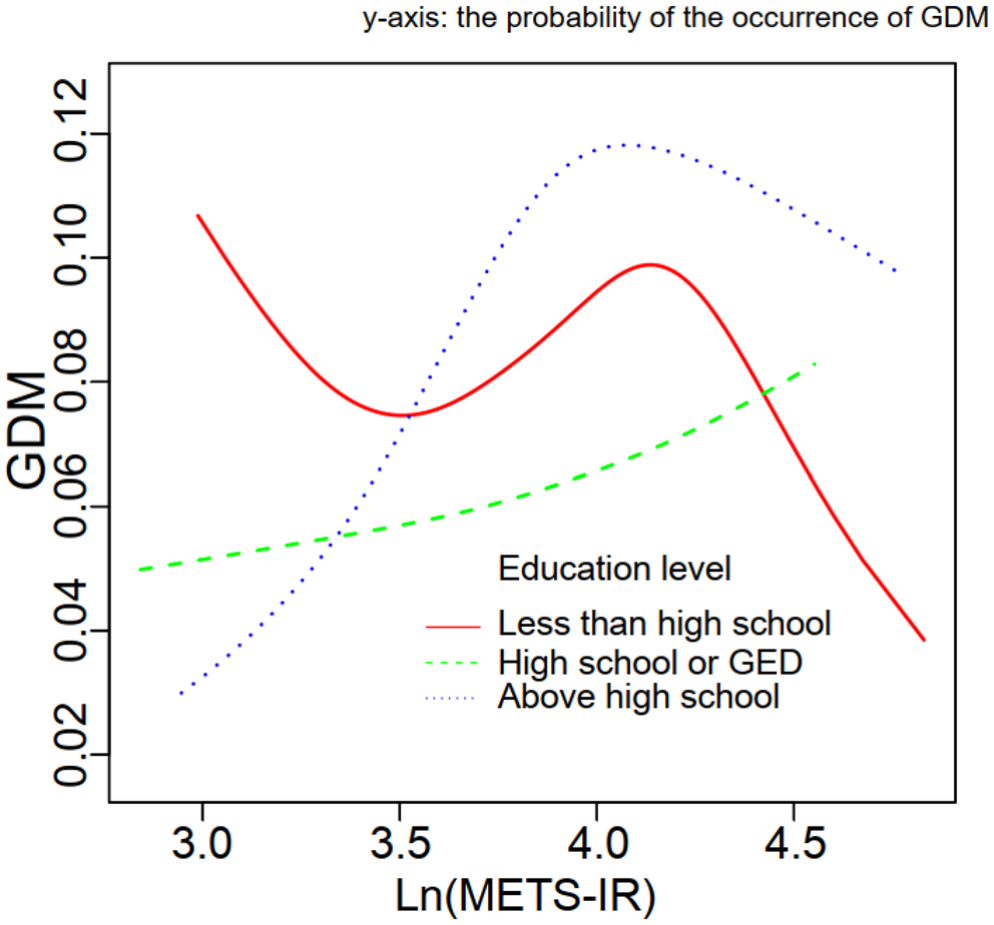
Smooth curve fitting for the relations between Ln(METS‐IR) and probability of GDM occurrence (%) in education levels.

Table 5 shows that the OR and 95% CIs of GDM for women above high school in the Q2, Q3 and Q4 quartiles were 2.05 (1.04, 4.02), 3.41 (1.72, 6.78) and 2.78 (1.55, 4.99), respectively. Additionally, Figure 4 shows a nonlinear relationship between the Ln(METS‐IR) and GDM in women above high school (*p* for nonlinearity = 0.011) and high school or GED (*p* for nonlinearity = 0.012).

**TABLE 5:** Regression analysis table stratified by educational level.

	Q1	Q2	Q3	Q4	*p* for trend	*p* for interaction
Education level	OR, 95% CI					0.0087
Less than high school	1.00	0.83 (0.33, 2.09)	1.13 (0.47, 2.74)	1.38 (0.51, 3.74)	0.3672	
High school or GED	1.00	0.57 (0.23, 1.42)	0.60 (0.24, 1.46)	0.70 (0.28, 1.75)	0.5360	
Above high school	1.00	2.05 (1.04, 4.02)	3.41 (1.72, 6.78)	2.78 (1.55, 4.99)	0.0002	

## 4 Discussion

The current study focused on whether METS‐IR can predict the occurrence of GDM using data from the nationally representative NHANES (2007–2018). A strong association was observed between higher Ln(METS‐IR) and elevated likelihood of GDM occurrence, particularly in individuals who underwent high school and above populations. Previous studies have also confirmed that a higher education level can indeed indirectly increase the risk of sedentary behaviour by influencing career choices, lifestyle evolution, health awareness, as well as social and cultural factors [16].

GDM is classified as a high‐risk pregnancy, prone to various adverse pregnancy outcomes such as stillbirth, preterm birth and macrosomia. Additionally, pregnant women with GDM and their offspring face an elevated risk of obesity, type 2 diabetes mellitus (T2DM) and cardiovascular diseases in the long term. Even more concerning is that, even with well‐controlled blood glucose levels, GDM patients still experience an increased risk of adverse perinatal outcomes and long‐term metabolic disorders for both pregnant women and their offspring. Lipogenesis is a critical biological process during pregnancy; however, recent studies have indicated that GDM patients exhibit significant lipogenesis dysfunction, leading to reduced adipose storage capacity and adipose dysfunction, which drives IR and accelerates the progression of GDM. Therefore, comprehensive consideration of biochemical indicator parameters related to adipose tissue metabolism can facilitate earlier and more accurate prediction of GDM, as well as safer and more effective clinical management.

Recent studies have revealed that METS‐IR can predict IR‐related diseases, such as T2DM, non‐alcoholic fatty liver disease, metabolic syndrome and others [17, 18, 19]. Specifically, researchers have found that METS‐IR is an independent prognostic indicator of IR in diabetes and non‐alcoholic fatty liver disease, closely associated with poor glycemic control outcomes in patients. Liu et al. found the association between METS‐IR and heart failure (HF) risk in adult diabetics within the United States [20]. In addition, elevated METS‐IR levels were associated with an increased risk of the presence and extent of atherosclerotic plaques and stenosis in non‐diabetic, older individuals in the PRECISE study [21]. Another study showed that METS‐IR was independently associated with the development of future diabetes in a non‐diabetic population. They pointed out that METS‐IR was a good predictor of diabetes, especially for women and individuals < 45 years old for predicting the future risk of developing diabetes at all times [22].

Compared to other IR indices, METS‐IR stands out for its insulin independence and superiority over TG/HDL‐C and TyG, making it a better predictor of DM and NAFLD, especially in younger populations [23]. Consistent with previous studies, the current study found a close correlation between METS‐IR and glycated HbA1c [24]. This study emphasises the efficacy of METS‐IR as a diagnostic tool for IR in patients with hypothyroidism, establishing it as a comprehensive alternative to HOMA‐IR rather than HbA1c and non‐based insulin level. In the fitted curve, we observed that as Ln(METS‐IR) increases, the incidence of GDM also increases when Ln(METS‐IR) is less than 4. However, when Ln(METS‐IR) exceeds the threshold of 4, the trend reverses. This may be due to the fact that when Ln(METS‐IR) surpasses 4, the metabolic disorder in the body becomes more severe, and the methods of blood glucose intervention increase accordingly. Therefore, the positive correlation between Ln(METS‐IR) and GDM is no longer obvious.

Some studies have reported that the correlation between METS‐IR and IR is stronger in certain populations, such as those with hypertension or who smoke. This study found that METS‐IR has a stronger correlation in Mexican‐American populations, those with high school education or higher, and individuals with hypertension. Consistent with previous research, this study also found that smoking increases the correlation between METS‐IR and GDM, although the difference was not statistically significant. Exercise is an important intervention for improving IR in pregnant women with GDM, and recent studies have also reported that exercise can improve maternal and fetal outcomes in GDM, preventing both short‐term and long‐term complications [25]. This study found a significant negative correlation between physical activity and METS‐IR, while sedentary time was significantly positively correlated with METS‐IR. Due to intrauterine exposure to hyperglycemia in GDM, the risk of having a large‐for‐gestational‐age (LGA) infant significantly increases. This study also found that an increase in METS‐IR increases the risk of delivering an LGA infant.

This study found that education level, rather than other factors, plays a significant role in the prediction of GDM by METS‐IR. Previous research on the correlation between education level and GDM has indicated that individuals with higher genetically predicted educational attainment have a lower risk of developing GDM [26]. Educational attainment has a potential causal protective effect on gestational diabetes, with obesity‐related risk factors playing a mediating role. Attention should be paid to the education level of women, as obese women with lower education levels may constitute a higher‐risk group for GDM compared to those with higher education [27]. Our study found that this hypothesis holds true for individuals with high school education or higher. However, for those with education levels below high school, the correlation trend between METS‐IR and GDM is more complex. This is likely because pregnant women with lower education levels have varying degrees of prenatal education, which disrupts the correlation trend between METS‐IR and GDM [28].

## 5 Conclusion

In summary, this study showed that the Ln(METS‐IR) was independently related to GDM in the general population. These findings suggest that the METS‐IR could be useful for identifying individuals at risk of poor clinical outcomes. Considering that the METS‐IR is easy and inexpensive to acquire, it could be a convenient tool for clinicians to stratify patient risks and plan preventive and treatment strategies.

## 6 Strengths and Limitations of the Study

Our study, which had a large research population and a long follow‐up period, allowed us to better understand the relationship between the METS‐IR and GDM. Furthermore, we used the smooth curve fitting and generalized additive models to analyse the nonlinear relationship between the METS‐IR and GDM. However, the study has some limitations, such as using self‐reported data for GDM and living habits, which may have memory bias. Additionally, we only collected the baseline value of the METS‐IR, which may not reflect changes in the METS‐IR during pregnancy.

## Author Contributions

**Hou Wenxuan:** conceptualisation, data curation, methodology, writing – original draft preparation. **Xu Lingyun:** data curation and methodology. **Tang Yujie:** software. **Zhang Ting:** methodology. **Han Zhen:** validation, visualisation and supervision. **Luo Xiao:** writing – reviewing and editing. **Yang Zhao:** writing – reviewing and editing.

## Ethics Statement

Before data from this study were included in the NHANES public database, all participants signed informed consent forms, adhering to the principles outlined in the Declaration of Helsinki, and were reviewed and approved by the NCHS Ethical Review Board.

## Conflicts of Interest

The authors declare no conflicts of interest.

## Supporting information


**Data S1.** Integrates the ROC curves of METS‐IR, FBG (fasting blood glucose), HbA1C (glycated hemoglobin) and TG (triglyceride) to compare the diagnostic efficiency of different indicators.

## Data Availability

The datasets generated and/or analysed during the current study are available in the (NHANES database) repository (http://www.cdc.gov/nchs/nhanes.htm).
