# Supplementary figures and images for: Association Analysis of Insulin Resistance Metabolic Score (METS‐IR) and Gestational Diabetes Mellitus: Based on National Health and Nutrition Examination Survey Database From 2007 to 2018
Source: Endocrinol Diabetes Metab. 2025 May 30;8(4):e70062. doi: 10.1002/edm2.70062 (PMC12124419; doi:10.1002/edm2.70062)

ROC curve for GDM

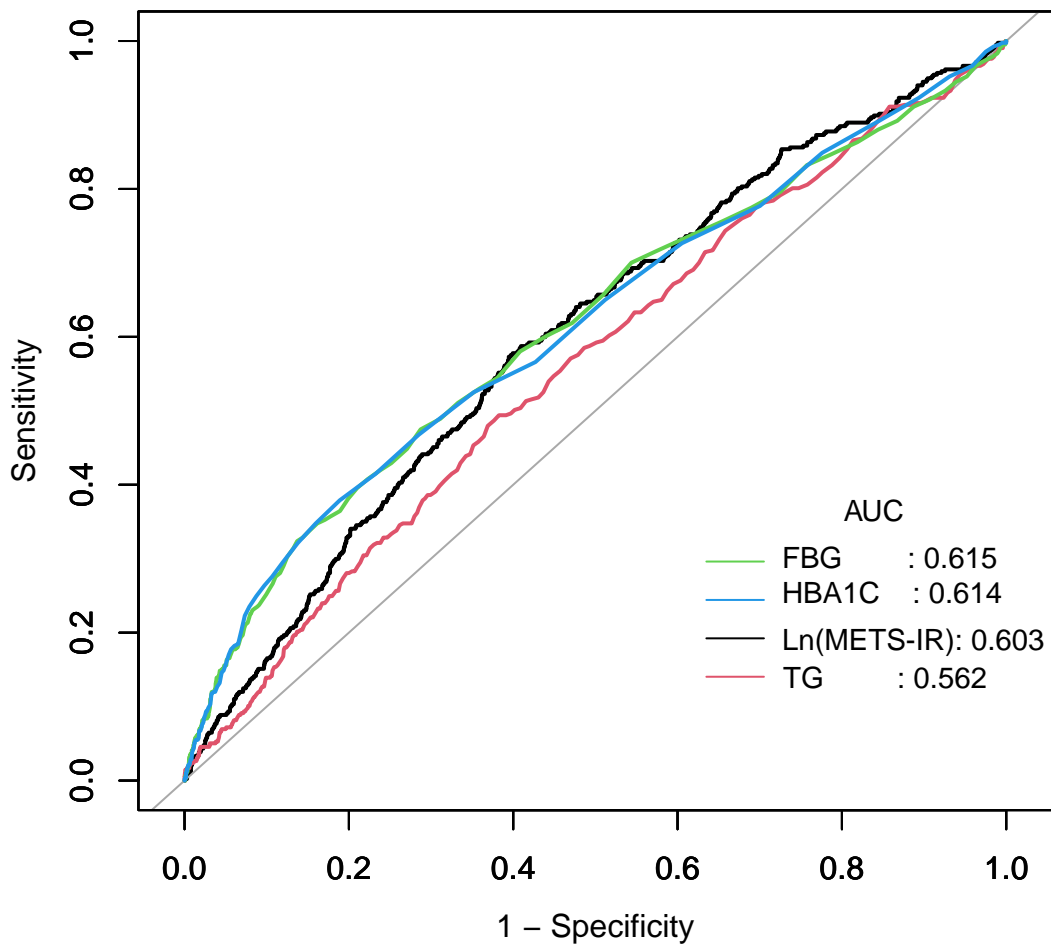

Supplement: Supplementary file 1 — Data S1. Integrates the ROC curves of METS‐IR, FBG (fasting blood glucose), HbA1C (glycated hemoglobin) and TG (triglyceride) to compare the diagnostic efficiency of different indicators. [file EDM2-8-e70062-s001.pdf]
